# UV induced ubiquitination of the yeast Rad4–Rad23 complex promotes survival by regulating cellular dNTP pools

**DOI:** 10.1093/nar/gkv680

**Published:** 2015-07-06

**Authors:** Zheng Zhou, Neil Humphryes, Patrick van Eijk, Raymond Waters, Shirong Yu, Rolf Kraehenbuehl, Edgar Hartsuiker, Simon H. Reed

**Affiliations:** 1 Institute of Cancer & Genetics, School of Medicine, Cardiff University, Heath Park, Cardiff, CF14 4XN, UK; 2 College of Biology, Hunan University, Changsha 410082, China; 3 New York University Department of Biology,1009 Silver Center, 100 Washington Square East, NY, USA; 4 Cambridge Epigenetix, Jonas Webb Building, Babraham Campus, Cambridge, CB22 3AT, UK; 5 North West Cancer Research Institute, Bangor University, Brambell Building, Bangor, LL57 2UW, UK

## Abstract

Regulating gene expression programmes is a central facet of the DNA damage response. The Dun1 kinase protein controls expression of many DNA damage induced genes, including the ribonucleotide reductase genes, which regulate cellular dNTP pools. Using a combination of gene expression profiling and chromatin immunoprecipitation, we demonstrate that in the absence of DNA damage the yeast Rad4–Rad23 nucleotide excision repair complex binds to the promoters of certain DNA damage response genes including *DUN1*, inhibiting their expression. UV radiation promotes the loss of occupancy of the Rad4–Rad23 complex from the regulatory regions of these genes, enabling their induction and thereby controlling the production of dNTPs. We demonstrate that this regulatory mechanism, which is dependent on the ubiquitination of Rad4 by the GG-NER E3 ligase, promotes UV survival in yeast cells. These results support an unanticipated regulatory mechanism that integrates ubiquitination of NER DNA repair factors with the regulation of the transcriptional response controlling dNTP production and cellular survival after UV damage.

## INTRODUCTION

DNA repair is central to the maintenance of genome integrity (1). A number of DNA repair mechanisms remove genetic damage by excising the lesion and replacing these regions of the DNA using the complementary strand as a template for repair synthesis. These mechanisms rely upon the regulated production of the building blocks of DNA, the deoxyribonucleotide triphosphates [dNTPs] (2,3). The cell strictly regulates the levels of dNTPs, which involves the production of dNTPs from NTPs, controlled by the ribonucleotide reductase (RNR). This process replenishes the dNTP pools following their incorporation into DNA.

Previously, we identified an E3 ubiquitin ligase comprised of the SOCS box domain Rad7 protein and RING domain Rad16 protein. These components of the yeast global genome nucleotide excision repair [GG-NER] pathway were found in complex with Elongin C and Cullin 3 forming a novel Cullin-RING-Ligase (CRL) referred to as the GG-NER E3 ligase (4). Together with transcription coupled nucleotide excision repair [TC-NER], these two pathways are responsible for repairing the non-transcribed and transcribed regions of the genome respectively (5). We revealed that the GG-NER E3 ligase complex enhanced UV survival via ubiquitination of the Rad4 protein. Rad4 is a member of the Rad4–Rad23 heterodimer, the yeast homologues of human XPC-hHRAD23B, which is the established DNA damage recognition factor involved in sensing damage during NER (6–9). Our results showed that the enhanced UV survival associated with the GG-NER E3 ligase complex was dependent on the UV induced ubiquitination of Rad4, but not its subsequent degradation (4,10,11). Of particular importance with regard to the present study is that the effect of the GG-NER E3 ligase on NER and UV survival is masked by the presence of Rad23. Moreover, we demonstrated that the effect of the E3 ligase on DNA repair is dependent on *de novo* protein synthesis (4). These findings revealed the presence of two redundant pathways contributing to DNA repair and UV survival: Pathway I dependent on the Rad23–19S proteasome interaction involving pre-existing proteins; and Pathway II requiring *de novo* protein synthesis, which is dependent on the UV induced ubiquitination of Rad4 within the Rad4–Rad23 complex by the GG-NER E3 ubiquitin ligase (4,12). Our results showed that inhibition of Pathway II by addition of cycloheximide, which inhibits translation of mRNA, causes UV sensitivity and inhibition of NER (4). This suggests that the GG-NER E3 ubiquitin ligase might regulate a component of the transcriptional response to DNA damage via the ubiquitination of the Rad4–Rad23 damage sensor.

To investigate this we initially conducted genome-wide microarray based gene expression profiling on different combinations of *RAD4* and *RAD23* deleted strains, to identify the genes whose expression is affected by the Rad4–Rad23 complex in the absence of UV damage. It is known that all Rad4 exists in a heterodimeric complex of Rad4–Rad23 (13–15). However, it is also established that the two single mutants have a variety of pleiotropic phenotypes, including differences in UV survival and lesion removal during NER (16,17). A Rad4 mutant is much more UV sensitive than a Rad23 mutant for example, and is also completely defective in NER, rather than the partial defect associated with *RAD23* deletion (16,18). Furthermore, Rad23 is far more abundant in the cell than Rad4, meaning that most of Rad23 does not exist in a complex with Rad4 (14,19). Indeed, Rad23 is also reported to be an accessory component of the 19S proteasome (15,20–23), and has a range of functions outside of NER, for example in spindle pole body duplication (24) and protein shuffling to the proteasome (23). In order to identify which of these genes are specifically regulated by the GG-NER E3 ligase, we introduced a GG-NER E3 ligase mutation referred to as *psocs*, which specifically fails to ubiquitinate Rad4 in response to UV (4). This enabled identification of the genes regulated by these complexes in response to DNA damage.

Our analysis revealed that ubiquitination of Rad4 by the GG-NER E3 ligase specifically regulates genes involved in the RNR pathway. In order to examine how Rad4–Rad23 might regulate the expression of these genes, we considered whether or not they bind directly to their promoters, since we noted that many of the genes we identified contained common regulatory elements. Chromatin IP showed that the Rad4–Rad23 complex associates with the promoter chromatin of some of these genes. Furthermore, in wildtype cells, UV induced DNA damage results in loss of occupancy of Rad4–Rad23 from these regulatory regions. This loss of occupancy is dependent on the ubiquitination of Rad4 by the GG-NER E3 ligase and is necessary for regulation of gene expression. This subsequently controls the expression of *DUN1* and some of its key downstream targets including the RNR pathway genes. This promotes the regulation of optimal dNTP levels in the cell by controlling the conversion of NTPs into dNTPs required for DNA repair synthesis (2,3). Finally, we show that the physiological role of this novel regulatory pathway is to ensure adequate production of UV induced dNTPs to enhance cell survival following DNA damage. Our results provide insight into a novel regulatory mechanism showing how NER factors regulate the transcriptional response that controls the production of dNTPs following DNA damage.

## MATERIALS AND METHODS

### Yeast strains and plasmids

Research Genetics parental strain BY4742, BY4742*rad23Δ*, BY4742*rad4Δ* and BY4742*rad7Δ* strains were obtained from Euroscarf. The double mutant *rad23Δrad4Δ* was derived from BY4742*rad23Δ* by replacing *RAD4* with a *HIS3* marker fragment. Creation of the Rad7 SOCS box mutation was described previously. Two point mutations were made, resulting in the amino acid substitutions L168A and C172A within the conserved SOCS box domain (4). The *RAD23* gene of BY4742*rad7*Δ was replaced by a *URA3* marker fragment to generate the double mutant *rad7*Δ*rad23*Δ. The triple mutant *rad7*Δ*rad23*Δ*sml1*Δ was derived from *rad7*Δ*rad23*Δ. Then pRS314 containing the *RAD7* gene and SOCS box mutated *RAD7* were introduced to *rad7*Δ*rad23*Δ*sml1*Δ respectively to produce the *pRAD7*Δ*rad23*Δ*sml1*Δ and *psocs*Δ*rad23*Δ*sml1*Δ strains. The data sets for the Rad4/Rad23 arrays can be found at:

http://www.ncbi.nlm.nih.gov/geo/query/acc.cgi?acc=GSE11871

The data sets for the psocs/rad23 arrays can be found at:

http://www.ncbi.nlm.nih.gov/geo/query/acc.cgi?acc=GSE23204

### *In vivo* cross-linking and sonication of chromatin extracts

Cells were grown to a density of 2-4 × 10^7^ cells/ml, and 2.8 ml of 37% formaldehyde was added to 100 ml of the culture medium (containing at least 2 × 10^9^ cells). The mixture was incubated at room temperature for 20 min with occasional swirling to allow efficient DNA and protein cross-linking. The cross-linking reaction was terminated by adding 5.5 ml of 2.5 M glycine to a final concentration of 0.125 M. Cells were collected by centrifugation and washed with ice-cold PBS buffer and ChIP lysis buffer. Cells were resuspended in 500 μl of ChIP lysis buffer supplemented with 12.5 μl of 20% sodium dodecyl sulphate (SDS) and 12 μl of 100x protease inhibitors. After 0.5 ml glass beads were added to this solution the mixture was vortexed at 4°C for 10–15 min. The cell lysate was carefully collected by centrifugation. Next, the cell lysate was sonicated by a Diagenode sonication system at the high output rate for 3–4 min (6–8 × 0.5 min on/0.5 min off cycle). The sonicated cell lysate was spun down at 13,200 rpm for 15 min at 4°C. The supernatant (chromatin extract) was finally transferred to a clean tube and stored at -80°C until further use.

### Chromatin immunoprecipitation (ChIP)

Protein A beads were washed twice with ChIP lysis buffer and then equilibrated with the same buffer supplemented with 0.1% BSA and 40 μg/ml single strand salmon sperm DNA for 3 h at 4°C. Next, 50 μl of chromatin extracts were added to 500 μl of ChIP binding buffer (i.e. ChIP lysis buffer supplemented with 0.25% SDS and 1x protease inhibitors), after which the solution was incubated with the equilibrated protein A beads. After removal of the protein A beads by centrifugation, the chromatin immunoprecipitation was carried out by adding 1–5 μl of antibody to this cleared solution and incubating at 4°C overnight. In the following step 20–30 μl of protein A beads slurry (ChIP lysis buffer washed twice) was added to the solution and incubated for 2–3 h at 4°C. The protein A beads were quickly spun and washed successively with ChIP lysis buffer, ChIP lysis buffer with 500 mM NaCl, LiCl solution and TE buffer.

The protein A beads were incubated with 250 μl elution buffer at room temperature for 10 min. Then, the supernatant was collected by centrifugation. The pellet was eluted again. The eluates were pooled and incubated at 65°C overnight to reverse the cross-linking.

Subsequently, the elution was treated with ribonuclease A and proteinase K and the DNA was purified by phenol/chloroform extraction or PCR purification kit (QIAGEN). In order to precipitate the DNA, 100 μg glycogen, 1/10 volume of 3 M sodium acetate (pH5.2) and 2 volumes of ethanol were added to the solution. The precipitated DNA was resuspended in 50–100 μl TE buffer and stored at -20°C.

### Determination of dNTP and NTP levels from yeast cells

Yeast cultures were grown in SD synthetic minimal medium (2% dextrose, 0.67% yeast nitrogen base) supplemented with 770 mg/l complete supplement mixture lacking histidine (CSM-his, Formedium) at 30°C to a density of 1 × 10^7^ cells/ml and collected by centrifugation at a 3.300 g. The untreated cells were kept aside while the cells for UV irradiation were resuspended in SD without supplement to a density of 2 × 10^7^ to 3 × 10^7^ cells/ml. This cell suspension was irradiated in 50 ml aliquots in Pyrex round dishes (135 mm diameter) in a CL-1000 Ultraviolet Crosslinker (UVP) with 20 J/m^2^ of UV-light (254 nm) at room temperature. The medium of the irradiated and unirradiated cells was supplemented by the addition of CSM-his. After treatment the cells were incubated in a shaker at 30°C for 3 h in the dark.

A total of 4 × 10^9^ to 1 × 10^10^ of unirradiated or irradiated cells were collected by filtration through AAWP nitrocellulose membranes (47 mm, 0.8 μm, Millipore). Nucleotide extraction and separation of dNTP and NTP on boronate columns was performed as described in (25). Eluates containing purified dNTPs were adjusted to pH 3.4 with 6 M HCl, loaded on a Whatman Partisphere SAX 5 μm HPLC column (250 × 4.6 mm) and isocratically eluted with 0.436 M potassium phosphate buffer (pH 3.35, 2.5% v/v acetonitrile). The aliquots for NTP measurement on HPLC were treated as described for dNTPs. Eluted nucleotides were detected with a Dionex PDA-100 Photodiode Array Detector based on their absorbance at 254 nm, and quantified by measuring peak heights using Chromeleon software and comparing with nucleotide standards.

## RESULTS

### The Rad4–Rad23 complex negatively regulates gene expression of a subset of UV responsive genes

We previously showed that ubiquitination of Rad4 by the GG-NER E3 ligase in response to UV radiation affected DNA repair and UV survival in a manner dependent on *de novo* protein synthesis (4). As described in the Introduction, this and other observations suggested a possible role of the Rad4–Rad23 complex in the regulation of gene transcription in response to DNA damage. Recently, multiple studies have confirmed a role for specific NER factors including yeast Rad23 and the human XPC-hHRAD23B complex in gene transcription (4,26–29). In order to identify genes that are regulated by the Rad4–Rad23 complex, we studied the effect on gene transcription using microarray gene expression analysis.

**Figure 1.**
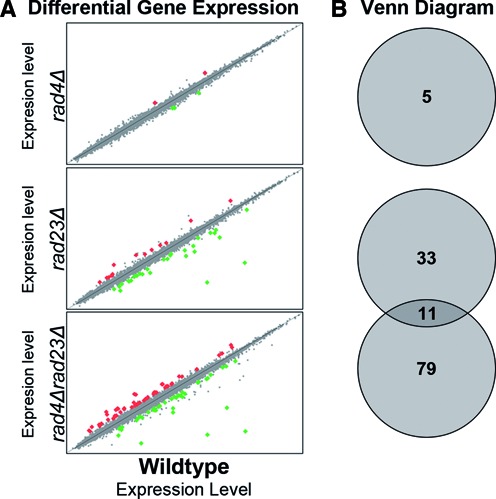
The Rad4–Rad23 NER factor has an effect on gene expression. (**A**) Significantly differentially expressed genes in *rad4Δ*, *rad23Δ* and *rad4Δrad23Δ* cells compared to wildtype cells in the absence of UV irradiation are plotted here. For each strain gene expression is compared to the wildtype control and plotted as a fold-change. Genes that did not display changed expression in the mutant backgrounds are in grey and plotted on the diagonal (*y* = *x*). Significantly upregulated genes are depicted in red while down regulated genes are shown in green. (**B**) Venn diagram of the significantly differentially expressed genes shown in panel (A) indicating the overlap between the changes in gene expression between the three different backgrounds tested.

To identify genes specifically regulated by the Rad4–Rad23 complex, we compared basal levels of gene expression between an untreated wildtype and double mutant strain deleted in both *RAD4* and *RAD23*. We found 90 genes whose expression was altered in the absence of the Rad4–Rad23 heterodimer (Figure 1, bottom panel). However, since it was previously reported that the deletion of *RAD23* has a broader effect on gene expression (27,28) and the additional roles of Rad23 in other cellular functions, we examined the effect of deleting only *RAD23* on gene expression (Figure 1, middle panel). We noted that loss of Rad23 resulted in altered expression of 44 genes of which only eleven are in common with the *rad4Δrad23Δ* double mutant strain. This indicates that 79 genes have altered expression specifically due to the loss of both Rad4 and Rad23. It also confirms a broader effect of Rad23 on gene expression outside of the Rad4–Rad23 complex (27). Finally, we examined gene expression changes in a *RAD4* deleted strain and found only 5 genes whose expression was altered, none of which are affected in the other strains. Importantly, this demonstrates that knocking out a major DNA repair pathway, even in the absence of DNA damage, does not cause indirect effects on gene transcription. The differential effect on gene expression we observed in the two single mutants could indicate that Rad4 and Rad23 affect gene transcription by two independent pathways. However, the pleiotropic effects associated with deletion of *RAD23* can also explain these observations. We cannot formally exclude the possibility that the effect on gene expression observed in the double mutant strain is due to the additive effect of two independent Rad4 and Rad23 pathways on transcription. However, since Rad4 and Rad23 always exist in a complex in the cell, coupled with our observation that only a small overlap in altered gene expression exists between the *rad23Δ* single and double mutant strains, we consider that the genes identified in the double mutant predominantly include those caused by loss of the Rad4–Rad23 complex from the cell.

**Figure 2.**
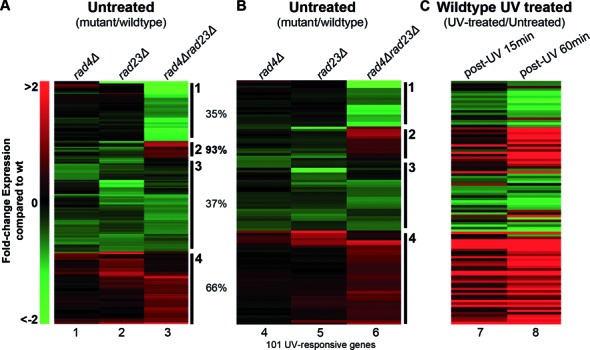
The Rad4–Rad23 NER factor affects gene expression of UV inducible genes. (**A**) Heat-map of the hierarchical clusters of the 205 affected genes in the mutant backgrounds expressed as a fold-change compared to wildtype expression. Indicated as percentages are the fraction of UV responsive genes within each cluster. (**B**) Heat-map of the hierarchical clusters of the 101 UV responsive genes from panel (A). (**C**) Gene expression changes of the equivalent genes as in B in UV irradiated wildtype cells relative to non-irradiated cells is depicted here.

Our observations are in keeping with the known pleiotropic effects of *rad4Δ* and *rad23Δ* single mutants. Following cluster analysis of the mutants, we noted that in untreated cells gene expression is *specifically* altered when both components of the Rad4–Rad23 complex are deleted (Figure 2A). In the double mutant strain we detect increased and decreased expression in the clusters (Figure 2A). Indeed, the genes in cluster 2 display increased expression in the *rad4Δrad23Δ* double mutant strain compared to either single mutant in the absence of UV damage (Figure 2A), suggesting that the Rad4–Rad23 complex causes repression of these genes in wildtype cells. We noted that 101 of the 205 genes in Figure 2A are UV responsive genes and we plotted these in Figure 2B. In Figure 2A cluster 2 stands out since 93% of these genes are UV responsive. Comparing the expression profile with the UV induced profile of these genes in wildtype cells (Figure 2C) demonstrates that the expression of these genes in unchallenged *rad4Δrad23Δ* double mutant cells mimics the expression profile of the same set of genes in UV irradiated wildtype cells. However, it should be noted that comparing the expression profile with the UV induced profile in wildtype cells demonstrates that the increased expression observed in *rad4Δrad23Δ* deleted cells in the absence of DNA damage does not reach the same level as the UV induced expression in wildtype cells (Figure 2B and C, compare lane 6 to 8). This suggests that whilst Rad4–Rad23 represses gene expression, derepression is necessary, but not sufficient, for the full level of expression observed after UV in wildtype cells.

**Figure 3.**
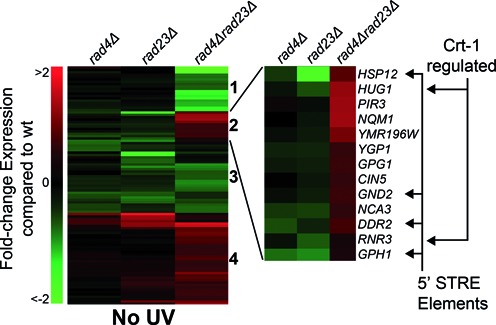
The Rad4–Rad23 NER factor affects gene expression of a subset of Crt1 and STRE regulated genes. Heat-map of hierarchically clustered genes in the *rad4/rad23* double and single mutants taken from Figure 2B. Cluster 2 is now highlighted to show the Stress Responsive Element (STRE) containing genes and Crt1 regulated genes affected in the mutant backgrounds in the right hand side of the figure.

**Figure 4.**
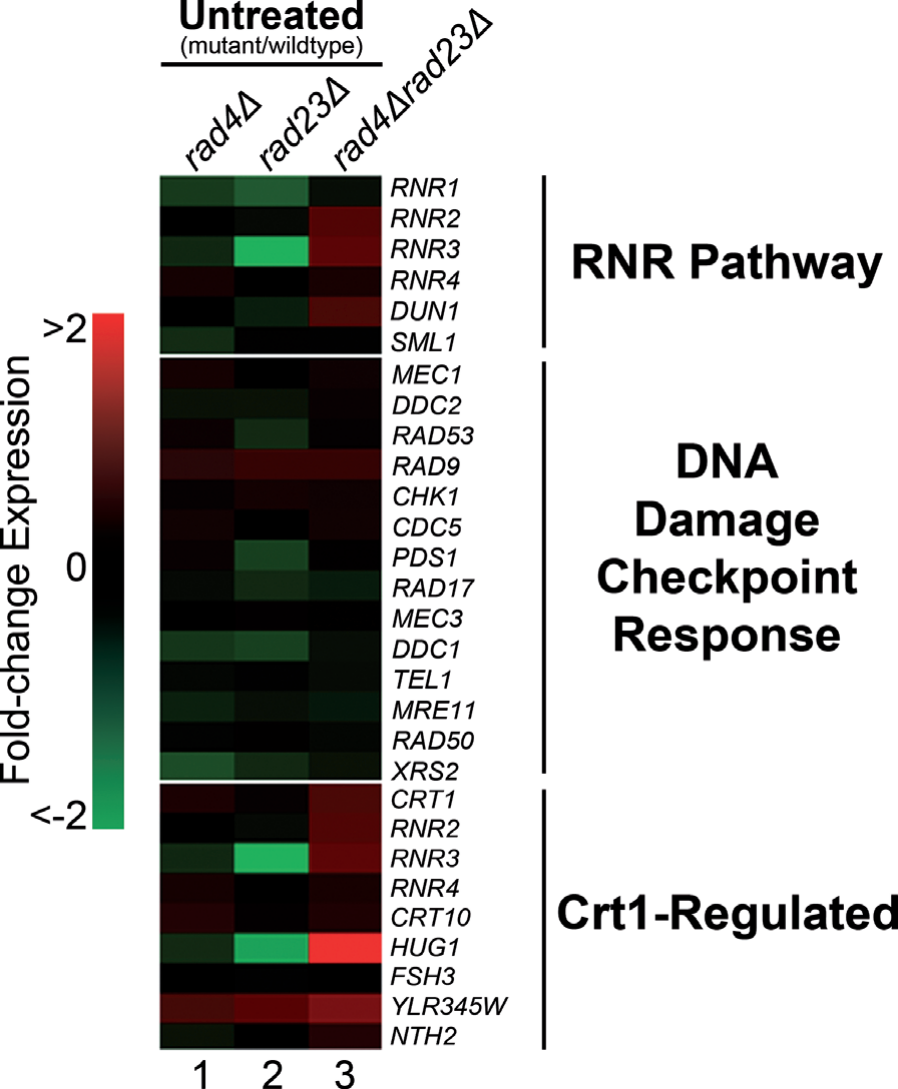
The Rad4–Rad23 complex is involved in gene regulation of RNR pathway and Crt1 regulated genes but not of genes belonging to the DDR. Differentially expressed UV responsive genes from the *rad4/rad23* data set are shown in this heat-map grouped by RNR Pathway, DNA damage checkpoint response and Crt1 regulated genes. Gene expression is displayed as the fold-change in relation wildtype cells (-2 to 2-fold).

**Figure 5.**
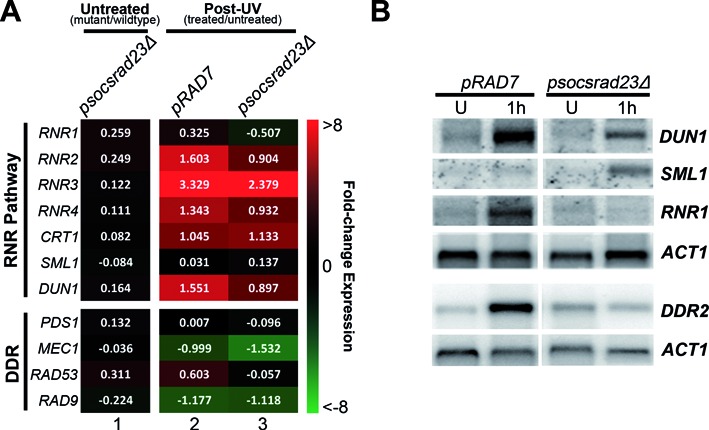
The GG-NER E3 ligase regulates UV induced gene expression in concert with Rad4–Rad23. (**A**) Quantitative representation of a selection of the genes shown in Figure 4 but now showing the UV induced changes to gene expression in the *pRAD7* and *psocsrad23Δ* strains compared to untreated cells. (**B**) Northern blot analysis of gene expression at the mRNA levels of candidate genes scored in the microarray analysis. RNA was extracted from the strains indicated and the blot was treated with probes for *DUN1, RNR1, SML1, DDR2* and *ACT1* as a loading control.

Importantly, many of the genes in cluster 2 contain either an STRE (Stress Response Element) or Crt1 regulatory sequence present in their promoters, as shown in Figure 3 (right panel). Crt1 is a transcriptional repressor of a set of UV induced cell-cycle checkpoint and DNA repair genes, including the RNR genes that control cellular dNTP synthesis. These genes become activated following derepression of the Crt1 protein, following its phosphorylation by Dun1 in response to DNA damage (30). Therefore, we extracted the RNR pathway and Crt1-regulated genes from the acquired gene expression data and compiled these into a single heat-map, together with other known members of the DNA damage checkpoint response, for comparison (Figure 4). These data are quantified for a selection of these genes in Figure 5A. A great deal is known about DNA damage signalling relating to the activation of this protein kinase cascade (30–32) (see Figure 8). However, little is known about the regulation of DDR gene expression. Our results show that increased gene expression of the STRE containing *DUN1* gene, the *RNR* genes and other Crt1-regulated genes can be detected in a *rad4Δrad23Δ* strain in the absence of UV, while other DNA Damage Checkpoint Response genes remain largely unaffected (Figure 4, lane 3). These observations suggest that in wildtype cells the Rad4–Rad23 heterodimer represses gene expression of a subset of STRE containing and Crt1 regulated, DNA damage inducible genes in the absence of UV irradiation.

It is noteworthy that the activation of these genes in *rad4Δrad23Δ* deleted cells is not caused indirectly by the constitutive activation of the DDR pathway in these cells. It is not possible for this to be the case because functional NER is required for DDR signalling (33). This is consistent with our microarray data (Figure 4), which indicates that the expression of DDR genes is unaffected in the *RAD4* and *RAD23* double mutant strain in the absence of DNA damage. This indicates that the altered gene expression observed in the double mutant strain involves the genes identified in the RNR pathway shown in Figure 4.

### The GG-NER E3 ligase activity is required for UV-induced DNA damage gene expression of Rad4–Rad23 regulated genes

As described earlier, the GG-NER E3 ubiquitin ligase complex functions in one branch of two parallel pathways involved in promoting efficient NER and UV survival. We showed previously that these two pathways, referred to as pathways I and II, have redundant functions, but can be differentiated by their dependence on either the Rad23–19S interaction or *de novo* protein synthesis, respectively (4). Consequently, in order to observe the effect of the E3 ligase dependent pathway II in NER and UV survival, it is necessary to delete the *RAD23* gene, which functions in pathway I and masks the role of the ligase in pathway II (see Figure 8 for further details) (4). As described previously, we mutated the GG-NER E3 ligase component Rad7 in its SOCS-box domain in a *rad23Δ* background (4). We next performed microarray gene expression analysis of the UV treated wildtype *(pRAD7)* and double mutant strains *(psocsrad23Δ)*. We analysed the UV induced change in gene expression of the RNR pathway, Crt1-regulated and DNA damage checkpoint response genes in each of these strains relative to unirradiated cells. The resulting heat-map is shown in Figure 5A and is expanded to show quantified transcript levels from the microarray data of the genes shown. The data for the *psocs* single mutant showing normal expression and UV induction are included in Supplementary Figure S1. The striking result from these analyses is that the RNR pathway and Crt1 regulated genes, including *DUN1* and *RNR2–4*, which show evidence for being regulated by the Rad4–Rad23 complex in the absence of UV (Figure 4, lane 3), are also dependent on the GG-NER E3 ligase activity for wildtype expression in response to UV radiation (compare Figure 5A, lanes 2 and 3, top panel). The UV induced expression of RNR pathway genes observed in wildtype (*pRAD7)* cells is significantly reduced in the E3 ligase defective *psocsrad23Δ* deleted strain to around 50–70% of wildtype. The *DUN1* and *RNR1* genes encode activators of the RNR pathway and the regulation of their expression in response to UV damage is important (34). In contrast, the DNA damage response genes, including *MEC1*, *TEL1* and *CHK1* in Figure 4, do not reveal a significant role for Rad4–Rad23 or the GG-NER E3 ligase in the regulation of expression of these genes (see Figure 5A, bottom panel). It appears that UV induced *RAD53* expression might be regulated by the GG-NER E3 ligase (Figure 5A, lane 3). However, it should be noted that increased *RAD53* expression is also observed in the *psocsrad23Δ* mutant in untreated cells (Figure 5A lane 1 and Supplementary Figure S1 lane 1). This indicates that *RAD53* expression is elevated in the absence of UV damage due to loss of Rad23. This likely explains the lack of *RAD53* induction in response to UV damage. The expression of other DDR genes such as *RAD9* and *RAD51*, are not significantly affected by Rad4–Rad23 or the GG-NER E3 ligase in response to UV (Figure 5A).

To confirm the microarray results, we performed northern blotting to detect RNA levels in the strains shown. Quantification of northern blot data for all genes listed in Figure 5A is shown in the Table 1. Figure 5B confirms the requirement for the GG-NER E3 ligase in upregulating both *DUN1* and *RNR1* in response to UV radiation as expected. This further emphasizes the role for Rad4–Rad23 and the GG-NER E3 ligase activity in the regulation of UV induced *RNR* and *DUN1* gene expression.

**Table 1.** Quantitative analysis of gene expression as determined by northern blotting for genes shown in figure 5A & B

Gene	*pRAD7* induction level	*psocsrad23Δ* induction level
*RNR1*	11.2	<1
*RNR2*	9.7	5.1
*RNR3*	19.5	10.2
*RNR4*	22.3	14.7
*CRT1*	24.3	26.0
*SML1*	1.8	9.3
*DUN1*	35.3	5.3
*PDS1*	<1	<1
*MEC1*	<1	<1
*RAD53*	7.0	<1
*RAD9*	<1	<1

We also noted that in *psocsrad23Δ* cells, UV irradiation results in elevated levels of *SML1* gene expression (Figure 5A and B). Sml1p is an inhibitor of the RNR enzyme complex and is degraded in response to DNA damage, thus activating the RNR enzyme (35,36). The increased expression of *SML1* in this strain following UV may further inhibit the conversion of NTPs to dNTPs by the RNR pathway.

### Rad4–Rad23 complex gene promoter binding inhibits transcription of UV responsive DDR genes

**Figure 6.**
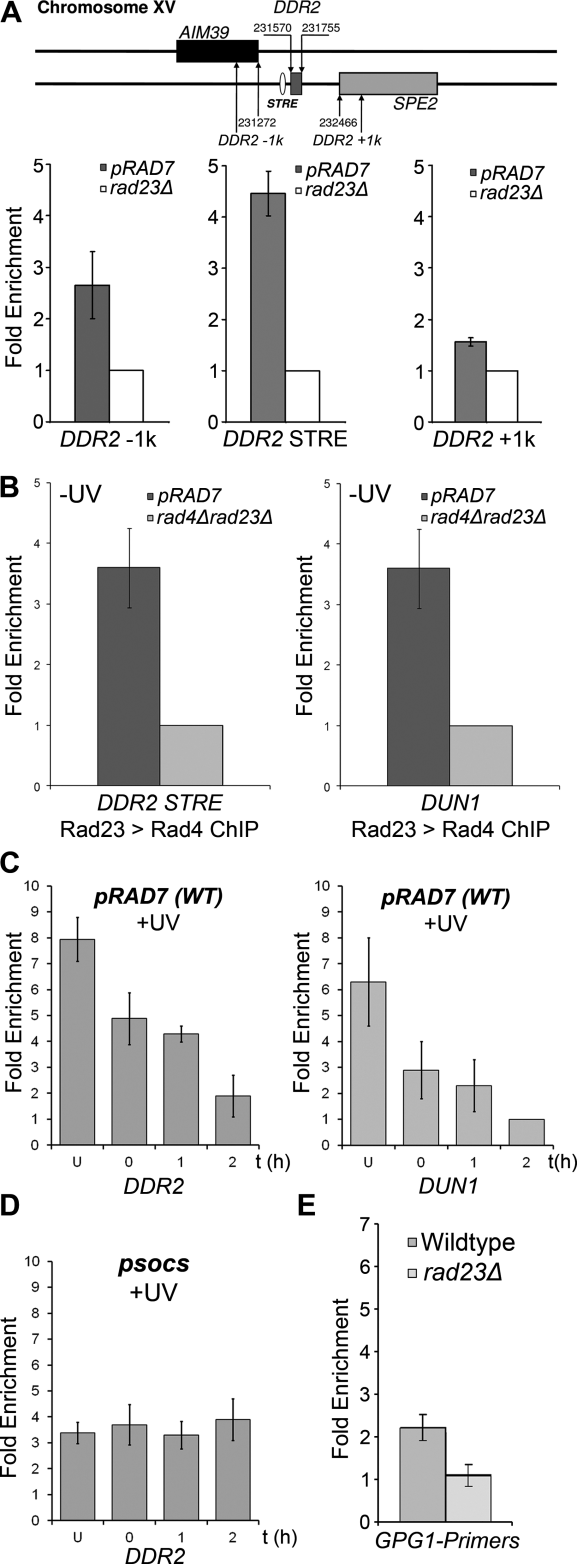
GG-NER E3 ligase dependent Rad4–Rad23 interaction with the promoter regions of the UV induced genes *DUN1* and *DDR2*. (**A**) ChIP-qPCR data of Rad23 interaction with the *DDR2* promoter. Rad23 antibodies were used to immunoprecipitate chromatin bound Rad23. Primer sets covering the STRE and areas 1kb upstream [DDR2–1k] and downstream [DDR2+1k] of the STRE of *DDR2* were used to quantify the relative enrichment of Rad23 (top panel). Rad23 protein occupancy at *DDR2* in absence of UV irradiation is shown in the lower panel, relative to the ChIP performed on *RAD23* deleted cells as a control. (**B**) Rad23 ChIP was subjected to a second round of IP detecting Rad4 as part of the complex interacting with the *DDR2* (right panel) and *DUN1* promoters (left panel). (**C**) Rad4–Rad23 interacts with the *DDR2* and *DUN1* promoters in response to UV. ChIPs were performed of untreated and UV irradiated chromatin from wildtype cells at different times after UV irradiation. (**D**) Rad4–Rad23 occupancy at the *DDR2* promoter in a GG-NER E3 ligase mutant. As panel (C) but for *psocs* cells showing no loss of occupancy of the Rad4–Rad23 complex from the *DDR2* promoter region. (**E**) Rad4–Rad23 does not interact with the *GPG1* gene promoter. The ChIP-qPCR experiment was performed using Rad23 antibody on wildtype and *rad23Δ* chromatin. qPCR analysis of the *GPG1* gene promoter was performed and shows no enrichment compared to background levels detected in a *RAD23* deficient strain. The Rad23 enrichment is relative to the background of the ChIP in *RAD23* deletion extracts set to unity. Data shown here are the average of three independent experiments with the standard deviation indicated by the error bars.

In an unirradiated *rad4Δrad23Δ* double mutant strain, increased gene expression observed for genes in cluster 2 mimics the UV induced increase in expression of these genes observed in wildtype cells (Figure 2B and C), albeit to a lesser extent. Intriguingly, recent evidence in mouse embryonic stem cells has revealed that XPC-RAD23B, the mammalian homologs of Rad4–Rad23, can regulate gene expression of specific developmental genes as a result of changes in the binding of the complex to transcription factors bound at regulatory elements in the promoter regions of these genes. This suggests a direct role for XPC-RAD23B in regulating gene transcription (29). These observations, together with the presence of STRE sequences in cluster 2 genes, prompted us to speculate that the Rad4–Rad23 heterodimer might also regulate UV induced gene transcription via direct or indirect binding of the complex to regulatory elements within the promoter regions of the UV responsive genes identified in Figure 3 (right panel). To examine this, we measured the occupancy of the Rad4–Rad23 heterodimer in the regulatory regions of the STRE element containing genes *DDR2* and *DUN1*, using chromatin immunoprecipitation (ChIP) and quantitative PCR. As shown in Figure 6A, we found that in unirradiated cells, the Rad4–Rad23 complex occupies the chromatin in the promoter region of *DDR2*. Figure 6A (top panel) indicates the location of three different sets of PCR primers in the proximity of the *DDR2* transcription start site. The lower panel of Figure 6A shows that the highest level of occupancy of Rad23 following ChIP is detected in the region of the STRE containing promoter element as measured by the *DDR2* STRE PCR primer set compared to two other primer sets located either 1kb upstream of the ORF [*DDR2–1k*] or downstream from the *DDR2* promoter [*DDR2+1k*].

We confirmed that Rad23 binds specifically at the STRE containing promoter region of the *DDR2* gene by examining Rad23 binding in the promoter region of the *GPG1* gene. *GPG1* expression is affected by Rad4–Rad23 as shown in Figure 3 (right panel), but does not contain an STRE element in its promoter sequence. No enrichment for Rad23 in the regulatory region of *GPG1* was detected (Figure 6E).

To examine the occupancy of both Rad4 and Rad23 in unirradiated cells at the promoter of *DDR2* and *DUN1*, we performed a double ChIP experiment, first performing chromatin immunoprecipitation using Rad23 antibodies, followed by a second IP using Rad4 antibodies. Figure 6B confirms the occupancy of both Rad4 and Rad23 at the promoter regions of the *DDR2* [left panel] and *DUN1* genes [right panel], indicating that they bind to the chromatin in the promoter region of these genes as a complex in the absence of DNA damage.

Next we examined the occupancy of the Rad4–Rad23 complex at the promoter regions of *DDR2* and *DUN1* in response to UV radiation. Figure 6C shows the loss of occupancy of Rad23 from the promoter region of both *DDR2* and *DUN1* following UV irradiation during a 2 h period. Our results demonstrate that the UV induced loss of occupancy of Rad4–Rad23 from the promoter regions of the UV responsive genes *DDR2* and *DUN1*, corresponds with an increased expression of these genes after exposure of cells to UV radiation as described earlier.

We then considered how the loss of occupancy of Rad4–Rad23 from the promoter regions of these genes is regulated in response to UV. Ubiquitination of Rad4 by the GG-NER E3 ligase plays an important role in NER and UV survival in a manner dependent on *de novo* protein synthesis (4). Furthermore, here we identify a role for the GG-NER E3 ligase in UV induced gene transcription (Figure 5). We speculated that this E3 ubiquitin ligase regulates the induction of UV responsive genes by controlling the occupancy of the Rad4–Rad23 complex at their promoter regions in response to DNA damage. To test this hypothesis, we measured Rad23 occupancy at the promoter of the *DDR2* gene in a mutant of the GG-NER E3 ligase. We examined events in strains either mutated in the SOCS-box domain of the Rad7 subunit or deleted for the *ELC1* subunit of the E3 ligase (data not shown). Both strains fail to ubiquitinate Rad4 in response to UV radiation (4). We show that, in contrast to wildtype cells, no loss of occupancy occurs for the Rad4–Rad23 from the *DDR2* promoter in response to UV damage in the *psocs* mutated strain (Figure 6D). Failure of these strains to ubiquitinate Rad4 in response to UV prevents the loss of occupancy of the Rad4–Rad23 complex from the promoter. Therefore, GG-NER E3 ligase activity promotes dissociation of Rad4–Rad23 from the promoter after DNA damage, enabling gene expression.

In summary, our results show that in wildtype cells the Rad4–Rad23 complex can act as a repressor of transcription of the *RNR* genes by binding to the chromatin at their promoter regions. Following UV irradiation, the Rad4–Rad23 complex is lost from the promoters of these genes in a GG-NER E3 ligase dependent fashion, facilitating the induction of gene expression.

### The GG-NER E3 ligase promotes increased levels of cellular dNTPs in response to UV damage

**Figure 7.**
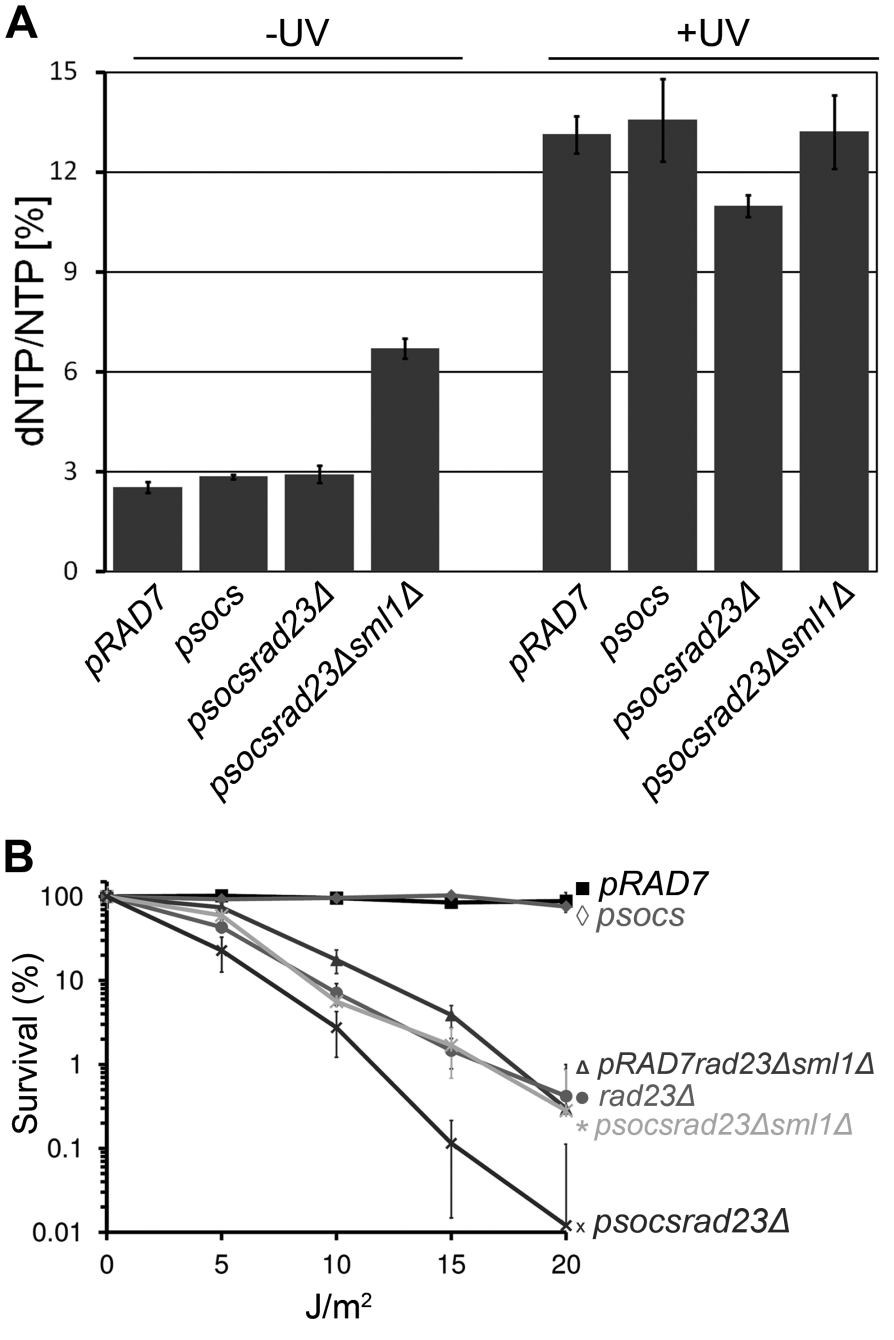
(**A**) The average dNTP/NTP increase in response to UV partly depends on the GG-NER E3 ligase in a *RAD23* deletion background. Concomitant deletion of *SML1* increases the dNTP pool to wildtype level. *pRAD7, psocs, psocsrad23Δ* and *psocsrad23Δsml1Δ* cells were collected and each dNTP/NTP ratio in the presence or absence of UV irradiation was determined by HPLC. The dNTP/NTP ratios depicted are an average of the dCTP/CTP, dTTP/TTP, dATP/ATP and dGTP/GTP ratios as a measure of the cellular dNTP pool for each strain in triplicate. The error bars represent the SEM. (**B**) The UV sensitivity due to altered gene expression in *psocsrad23Δ* mutants can be rescued by derepressing dNTP synthesis. UV survival curves of *psocsrad23Δ* strains rescued by deleting *SML1* are displayed here. Cells of the strains indicated were grown to log-phase and treated with increasing doses of UV radiation. Survival is quantified as colony-growth on YPD plates 2–3 days after UV treatment.

The results described suggest that the GG-NER E3 ligase regulates the expression of RNR pathway genes, which control cellular dNTP pools. To ascertain whether this is the case, we measured the cellular dNTP levels of wildtype *(pRAD7)* and GG-NER E3 ligase mutated (*psocsrad23Δ*) strains. We measured average dNTP/NTP ratios, which are indicative of the activity of the RNR pathway, and as expected found similar dNTP levels for the wildtype (*pRAD7)* and E3 ligase mutated *psocs*, and *psocsrad23Δ* strains in the absence of UV irradiation (Figure 7A, −UV), in agreement with our microarray gene expression data (Figure 5A, lane 1). As anticipated, following DNA damage after UV irradiation, the average increase in overall dNTPs 2 h later is readily detectable in both *pRAD7* and *psocs* strains (Figure 7A, +UV). However, in contrast, the *psocsrad23Δ* double mutant strain exhibits lower levels of UV induced dNTP/NTP ratios. To determine whether constitutively activating the RNR pathway by deleting the *SML1* inhibitor of the RNR complex might rescue this phenotype, we created the *psocsrad23Δsml1Δ* strain. As expected we found that the dNTP/NTP ratio in the absence of UV is higher in this strain due to loss of inhibition of the RNR complex (Figure 7A, −UV). Significantly, we observed that the reduced dNTP levels observed in *psocsrad23Δ* cells after UV irradiation are rescued to wildtype levels when the RNR pathway is constitutively activated in the *psocsrad23Δsml1Δ* strain (Figure 7A, +UV). Therefore, the altered regulation of gene expression observed in the *psocsrad23Δ* strain affects the UV induced regulation of the RNR pathway, resulting in reduced dNTP levels observed in response to DNA damage.

### The GG-NER E3 ligase promotes UV survival by regulating cellular dNTP pools

In order to examine the physiological role of UV induced dNTP pool regulation, we investigated its effect on UV survival. As shown previously, the UV sensitivity of the double mutant *(psocsrad23Δ)* is significantly greater than the *rad23Δ* single mutant, while the single *psocs* mutant is not UV sensitive (4). Based on our results we reasoned that the extreme UV sensitivity of *psocsrad23Δ* cells could be caused in part by the failure to upregulate the expression of the RNR genes, which results in reduced dNTP production following UV irradiation. Therefore, as described in the previous section, we attempted to rescue the UV sensitivity of the *psocsrad23Δ* strain by constitutively increasing the cellular dNTP pools. Therefore we measured UV survival in the *psocsrad23Δsml1Δ* strain. Figure 7B shows that upregulation of dNTP pools in this strain does indeed rescue the UV sensitivity of the *psocsrad23Δ* double mutant. This result confirms that a defective RNR pathway in the absence of GG-NER E3 ligase activity causes the increased UV sensitivity of *psocsrad23Δ* cells.

## DISCUSSION

Cells exposed to DNA damaging agents activate a DNA damage response that allows cells to halt cell-cycle progression, permitting time to repair the damage. Signalling cascades involving post-translational modifications of key regulatory proteins and an extensive DNA damage-induced gene expression programme are processes that underpin this response. It is the interplay between these networks that provide the cell with the opportunity to successfully complete DNA repair and enhance its survival following DNA damage. Current understanding of the DDR and the activation of signalling have mainly been concerned with signal processing within the cascade of protein kinases. However, much less is known about the regulation of gene expression, which ensures the timely production of the proteins that comprise the DDR. Here we describe a mechanism whereby core NER factors directly inhibit the expression of specific DDR genes until their UV-induced removal from the regulatory regions of these genes, ensuring the appropriately timed production of key DDR proteins and the dNTP pools they regulate.

**Figure 8.**
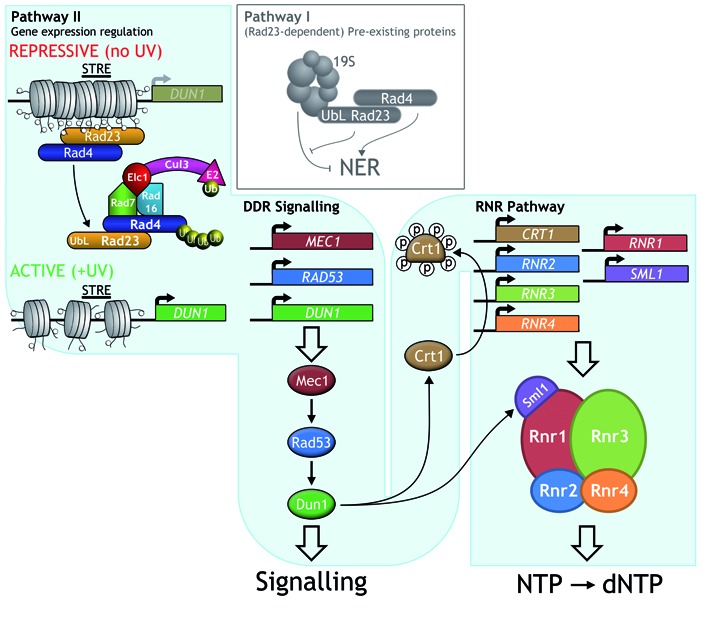
A model for Rad4–Rad23 and GG-NER E3 ligase function in DDR signalling and dNTP synthesis. The top left side and top middle panel of the figure shows the redundant two-pathway NER system as previously described (4). Pathway I involves Rad23 and the 19S proteasome that control NER independently of *de novo* protein synthesis, while Pathway II involves Rad4 ubiquitination by the GG-NER E3 ligase that regulates the gene expression of *DUN1* and *DDR2* and other genes described in this study. Ubiquitination of Rad4 (left panel) drives dissociation of the Rad4–Rad23 complex from the promoter region of STRE containing genes. *DUN1* is shown as an example. This loss of occupancy promotes increased gene expression. This example is specific for the STRE containing *DUN1* gene and other genes from cluster 2 in Figure 3. Rad4–Rad23 binding in the vicinity of the STRE may be direct or indirect. The central signalling cascade of Mec1-Rad53-Dun1 is represented in the middle section, including the *RNR* pathway as an important end-point in the bottom right panel. In response to DNA damage Crt1 is hyperphosphorylated by activated Dun1. Derepression of the *RNR* genes by Rad4–Rad23 and Crt1 results in enhanced expression of these genes and subsequent increase of the cellular dNTP pools. The transcription response of *DUN1* and *RNR1–4* provides downstream substrates for the DDR signalling pathway. How pathway II is activated in response to DNA damage remains to be determined.

Using microarray gene expression profiling to determine the effect of Rad4–Rad23 and the GG-NER E3 ligase on gene expression in response to DNA damage, we identified a group of genes which are upregulated in response to UV radiation in wildtype cells, and that require Rad4–Rad23 for their inhibition in the absence of DNA damage. This suggests that in undamaged wild type cells, Rad4–Rad23 acts as a repressor of these genes, which become activated in response to exposure of cells to UV radiation. Examination of this group of genes revealed STRE containing and Crt1 regulated genes of the RNR pathway. Using a similar approach, we also identified which of these genes are specifically regulated by the GG-NER E3 ligase via its ubiquitination of Rad4 in response to UV radiation. In summary, the UV induced control of RNR pathway gene expression is misregulated in the absence of this E3 ubiquitin ligase activity due to reduced *DUN1* and *RNR2–4* gene expression and increased expression of *SML1;* the RNR enzyme complex inhibitor (Figure 8).

We next considered how the Rad4–Rad23 complex controls gene expression by examining its binding to chromatin at the promoter regions of these genes. To investigate this, we measured the occupancy of the Rad4–Rad23 complex at the promoters of these STRE containing genes, including *DUN1* and *DDR2*, using ChIP and qPCR. Because our experiments examine protein interactions in chromatin, we are not able to determine whether the Rad4–Rad23 complex binds directly to the STRE in the gene promoter, or indirectly to the element via the binding of another transcriptional regulator present in the vicinity of the STRE. It is noteworthy that the interaction of the mouse XPC-RAD23B complex to the promoters of the Oct4-Sox2 regulated genes is thought to occur indirectly via protein-protein interaction between the NER complex and Oct4-Sox2 (29). Nevertheless, the results of our experiments explain the function of the GG-NER E3 ligase in controlling the Rad4–Rad23 dependent gene expression of these genes. In the absence of UV damage, we found that Rad4–Rad23 binds to the STRE containing promoter regions and in response to UV radiation, ubiquitination of Rad4 by the GG-NER E3 ligase promotes the dissociation of Rad4–Rad23 from the gene promoters. Loss of occupancy of Rad4–Rad23 from the promoter is necessary but not sufficient for full activation of the UV induced genes investigated.

In addition to *DUN1* and *DDR2* we noted that other genes have similar expression profiles, raising the possibility that these genes also influence dNTP synthesis in a manner yet to be determined. Our findings are consistent with the model shown in Figure 8 in which Pathway II regulates the expression of genes including *DUN1*, a key regulator in the control of the RNR pathway downstream of the central spine of the Mec1-Rad53 signalling pathway. The GG-NER E3 ligase-mediated UV-induced gene transcription regulation promotes the production of the downstream factors required by the DDR, by increasing *DUN1* and *RNR2–4* gene expression following DNA damage. This ensures sufficient production of the protein targets for the upstream members of the DDR signalling pathway to act on (see Figure 8). Activation of the DDR signalling pathway, including Rad53 phosphorylation, is known to be dependent on some level of functional NER. Significantly, the DDR can be triggered in a *rad7Δ* GG-NER mutant, which is partially defective in NER, but not in *rad2Δ or rad14Δ* deleted cells where NER is completely defective (33). This result is consistent with our observations, which show that the gene expression regulated by the GG-NER E3 ligase involves genes acting downstream of Mec1 and Rad53. Furthermore, our data also confirm previous findings (33) that gene expression of the DDR pathway is not constitutively activated in the absence of UV damage when NER function is deleted (see Figure 5A, left panel). This eliminates the possibility that our observations are due to the constitutive activation of the DDR pathway in the absence of damage in *RAD4, RAD23* deleted cells.

Our analysis focuses on the role of Rad4–Rad23 in repressing a subset of UV responsive genes. However, we also identified a group of UV inducible genes that require Rad4–Rad23 for their activation (cluster 1, Figure 2A and B), indicating that the complex may also be an activator of gene expression, consistent with a recent report (28,29). This implies that Rad4–Rad23 may have both positive and negative effects on gene expression.

Our results provide a novel mechanism by which the nucleotide excision repair pathway integrates with the DDR, and demonstrates how core NER factors also regulate the production of dNTPs, the raw materials required for enhanced UV survival. The common initiating event for DDR induction is DNA damage sensing that triggers signalling. Here, we show a mechanism for DNA damage induced gene expression of DDR related genes that can stimulate dNTP synthesis independently from the central kinase cascade of the DDR pathway. Finally, we demonstrate that the physiological significance of this pathway involves increasing dNTP synthesis in response to UV damage, promoting enhanced UV survival.

Future studies will focus on two key areas: firstly, how the GG-NER E3 ligase activity is initiated in response to UV irradiation and secondly, to uncover the mechanism behind the redundancy observed between pathway I and II in order to define the overlapping function of these pathways. One intriguing possibility is that in the presence of Rad23, pathway I may also regulate dNTP pool levels, but in a manner independent of *de novo* protein synthesis.

In conclusion, our results show that GG-NER E3 ligase induced gene expression changes following DNA damage serve to enhance dNTP synthesis, which promotes survival in response to UV radiation.

## ACCESSION NUMBERS

The data sets for the Rad4/Rad23 arrays have accession number GSE11871. The data sets for the psocs/rad23 arrays have accession number GSE23204.

## Supplementary Material

SUPPLEMENTARY DATA
