# Supplementary material for: UV induced ubiquitination of the yeast Rad4–Rad23 complex promotes survival by regulating cellular dNTP pools
Source: Nucleic Acids Res. 2015 Jul 6;43(15):7360–70. doi: 10.1093/nar/gkv680 (PMC4551923; doi:10.1093/nar/gkv680)
Supplement: SUPPLEMENTARY DATA [file supp_43_15_7360__index.html]

UV induced ubiquitination of the yeast Rad4–Rad23 complex promotes survival by regulating cellular dNTP pools — UV induced ubiquitination of the yeast Rad4–Rad23 complex promotes survival by regulating cellular dNTP pools — SUPPLEMENTARY DATA 

# UV induced ubiquitination of the yeast Rad4–Rad23 complex promotes survival by regulating cellular dNTP pools

## SUPPLEMENTARY DATA

- SUPPLEMENTARY DATA
