# Supplementary material for: UV induced ubiquitination of the yeast Rad4–Rad23 complex promotes survival by regulating cellular dNTP pools
Source: Nucleic Acids Res. 2015 Jul 6;43(15):7360–70. doi: 10.1093/nar/gkv680 (PMC4551923; doi:10.1093/nar/gkv680)
Supplement: SUPPLEMENTARY DATA [file supp_gkv680_nar-01192-x-2015-File010.docx]

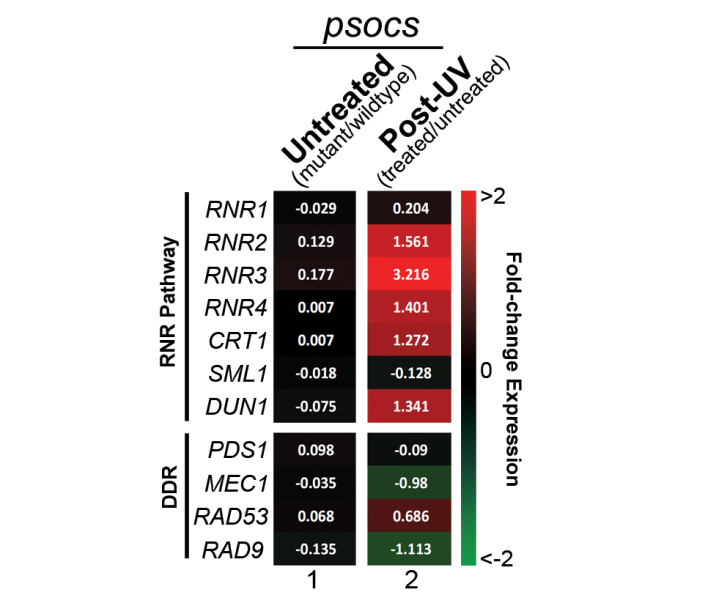


Figure S1 – The GG-NER E3 ligase SOCS box mutation does not affect basal- and DNA damage induced gene expression of Rad4-Rad23 regulated genes by itself. Quantitative representation of a selection of the genes shown in Figure 5 but now showing the UV induced changes to gene expression in the *psocs* strain comparing expression between mutant and wildtype cells (left panel) and treated and untreated *psocs* cells (right panel).
